# Whey Proteins and Immunity: Mechanisms Underlying Immune System Reinforcement and Protection Against Viral and Bacterial Infections

**DOI:** 10.3390/nu18111770

**Published:** 2026-05-30

**Authors:** Jean-François Lesgards

**Affiliations:** Independent Researcher, 13397 Marseille, France; jf.lesgards@gmail.com

**Keywords:** whey proteins, immunity, antiviral, antimicrobial, anti-inflammatory, gut microbiota

## Abstract

This review aims to examine the immunological, anti-inflammatory, antiviral, and antibacterial activities of key whey and milk proteins, specifically lactoferrin, glycomacropeptide, β-lactoglobulin, α-lactalbumin and their derived peptides, particularly lactoferricin and lactoferrampin, highlighting their potential as preventive or therapeutic agents. Whey and dairy products represent complex biological matrices that, beyond their high nutritional value, serve as reservoirs of bioactive proteins and peptides with documented health-promoting properties. It has been reported that certain whey proteins (WPs) and whey-derived peptides may contribute to improvements in both innate and adaptive immunity, exert direct antiviral and antibacterial effects while also modulating host defenses through immunoregulatory, antioxidant, and anti-inflammatory activities. These mechanisms contribute not only to enhanced resistance against viral pathogens but also to maintaining intestinal homeostasis and microbiota balance, both of which are critical during infection. In recent years, particularly in the context of the COVID-19 pandemic, natural bioactive compounds derived from whey, and, more broadly, milk, have attracted increasing attention as potential adjuncts or alternatives to conventional antivirals, with reported activity not only against SARS-CoV-2, influenza but also other viral and microbial infections. Despite encouraging in vitro and in vivo evidence, clinical validation remains limited, and the antiviral and immunomodulatory effects of WPs still require deeper mechanistic clarification. Future research should focus on identifying molecular targets, as well as characterizing the pharmacokinetics and safety profiles of WPs and WP peptides across diverse clinical settings. At the same time, attention should be given to optimizing their application as nutraceuticals or functional dairy ingredients.

## 1. Introduction

The innate immune system provides rapid, nonspecific defense through barriers, cytokines, and cells such as macrophages, dendritic cells, and NK cells, while the adaptive system generates antigen-specific and long-lasting protection via T and B lymphocytes, forming an integrated network essential for host defense [1].

Bioactive milk components, particularly whey proteins (WPs), have emerged as molecules of interest due to their immune-modulating, antiviral, and anti-inflammatory properties [2,3]. Other natural compounds, including vitamin D, zinc, selenium, polyphenols, and balanced diets rich in fruit, vegetables, and omega-3 fatty acids, support immune and antioxidant defenses, whereas malnutrition and obesity increase susceptibility to severe infections [4,5,6,7,8,9,10].

Since the beginning of the COVID-19 crisis, public interest in hygiene, immunity, and inflammation has increased. Global mortality remained below 0.1% of the population, with CFR estimated below ~0.5%, indicating >99.5% survival [11,12]. COVID-19 vaccines have been reported to reduce severe outcomes, with ongoing evaluation of their global impact and documented adverse effects [11,13]. Dairy intake, particularly low-fat products, has been associated with reduced COVID-19 severity and hospitalization [14,15]. More broadly, diets rich in fruits, vegetables, and omega-3s, and low in processed foods, may reduce inflammation and infection risk [16,17,18], whereas malnutrition and obesity impair immunity and increase COVID-19 severity [19].

Overall, in addition to conventional medical treatments, optimizing nutrition and incorporating functional components such as whey proteins, may offer valuable prophylactic and therapeutic support against infectious and inflammatory diseases by strengthening immune function and helping to regulate oxidative and inflammatory stress [20,21,22].

Whey, a by-product of cheese and curd manufacturing formerly considered a waste product, has been reported to show antiviral and antibacterial properties, including against SARS-CoV-2 and other pathogenic microorganisms [23,24]. Whey is a milk-derived protein complex now considered a functional food with multiple potential health benefits [25,26]. Its main bioactive components include β-lactoglobulin (β-Lg, 45–60%) and α-lactalbumin (α-La, 15–25%), as well as immunoglobulins (IGs, 10–15%), glycomacropeptide (GMP, up to 25%), serum albumin (SA, ~10%), lactoferrin (Lf, ~1%), and trace amounts of lactoperoxidase (<1%). Historically, whey has also been valued for its medicinal properties, being mentioned since Ancient Greece by Hippocrates and used throughout the Middle Ages for wound care, vitality, and various ailments [27].

Whey protein is commercially available in several forms, including whey protein concentrates (WPCs, 25–80% protein), whey protein isolates (WPIs, ≥90%), hydrolyzed whey proteins, and purified fractions such as β-lactoglobulin (β-Lg), α-lactalbumin (α-La), and protein-derived peptones [28]. High-quality WPCs and WPIs obtained through low-temperature membrane filtration may better preserve sensitive amino acids, including sulfur amino acids (methionine, cysteine) and tryptophan. In contrast, some industrial processing methods (e.g., heat or ion exchange treatments) can alter protein structure and reduce the integrity of certain residues [29,30,31]. These amino acids and bioactive peptides are considered relevant for immune function and may contribute to host defense against viral and bacterial infections, including influenza and coronaviruses [32,33].

This review primarily aims to provide a broad and detailed overview of the cellular and molecular pathways and mechanisms through which whey proteins and their derived peptides may strengthen immune defenses and regulate immuno-inflammatory responses.

Beyond these insights, this review also aims to raise awareness of the potential value of nutritional and functional food strategies, alongside conventional treatments, in supporting the prevention and management of infectious and immune-related diseases, while emphasizing the need for further clinical research in this field.

## 2. Effects of Whey Protein Intake on Innate and Adaptive Immune Cells

### 2.1. Glutathione Synthesis Improvement Effect on Immune Cells

Whey proteins have been shown to enhance both innate and adaptive immunity [34]. This effect may be largely mediated through their role in glutathione (GSH) synthesis and regulation. Their high content of cysteine and methionine provides essential precursors for GSH production, which is crucial for maintaining intracellular redox balance and immune cell function [35,36]. Indeed, a reduced GSH state promotes proliferative signaling, explaining the mitogenic effect of whey proteins on leukocytes, particularly lymphocytes, whereas oxidative conditions favor stress responses and apoptosis [35,36,37,38,39].

In the innate immune system, whey proteins, particularly β-lactoglobulin and α-lactalbumin, have been shown to enhance neutrophil function [40]. Mechanistically, GSH supports phagocytic cells such as monocytes and macrophages by improving oxidative killing mechanisms and may also contribute to natural killer (NK) cell activity through maintenance of redox homeostasis [40,41,42,43].

Natural killer (NK) cells are particularly key innate immune effectors involved in antiviral defense and immunomodulation, and, together with T cells, contribute to tumor cell elimination [44,45]. In a 12-week trial in patients with chronic hepatitis, supplementation with 12–20 g/day of non-heated whey proteins increased both hepatic GSH and NK cell activity, indicating improved redox status and immune recovery, alongside clinical improvement without major adverse effects [46].

In the adaptive immune system, in vivo and in vitro evidence also supports these effects. Consistently, whey proteins also stimulated murine splenic lymphocyte proliferation in vitro [40] and improved T lymphocyte activation and function as reflected by increased GSH-dependent immune responsiveness [35,38,47].

Finally, modulation of GSH metabolism may also contribute to the selective effects of WPC on tumor cells, which often exhibit elevated intracellular GSH levels, thereby increasing their susceptibility to chemotherapy [48].

### 2.2. Effect of Whey Intake on Innate Immune System

Bovine WPCs (500 mg/mg β-lactoglobulin, 15 mg/mg α-lactalbumin, 100 mg/mg GMP, 30 mg/mg LF, 20 mg/mg bovine IgG, 9.6 mg/mg bovine serum albumin) have been shown to prime human neutrophils by enhancing their functions and stimulating strong production of the anti-inflammatory cytokine IL-1Ra [40,49]. Its active components, beta-lactoglobulin and alpha-lactalbumin, act additively to reproduce these effects and can shift the balance toward anti-inflammation (high IL-1Ra:IL-1β ratio) via MAPK and NF-κB pathways, suggesting immunomodulatory and host defense benefits.

Additionally, as previously mentioned, WPs can reinforce NK cell function. Non-heated whey proteins (12–20 g/day for 12 weeks) improved immune function in some chronic hepatitis B patients by increasing NK cell activity and IL-2 levels, while also reducing ALT and oxidative stress [46]. Similarly, a randomized, double-blind trial (*n* = 80) showed that 8-week supplementation with 4.2 g/day fermented whey protein significantly increased NK cell activity across all effector:target ratios (10:1 to 1.25:1) compared with placebo, with these changes accompanying higher IL-12 levels and significant correlations between ΔIL-12 and NK activity (r = 0.571, *p* < 0.001) as well as pre-albumin (r = 0.435, *p* = 0.006) [50]. In line with these results, a metabolomics sub-study (*n* = 50) confirmed increased NK cell activity together with higher Lp-PLA_2_ activity (*p* = 0.021) and IL-1β levels (*p* = 0.001), while also identifying 10 significantly altered metabolic pathways, especially amino acid and sphingolipid metabolism, linking these immune effects to systemic metabolic reprogramming [51].

Also, in suckling Wistar rats, WPC (0.2 g/kg/day) selectively enhanced mucosal innate immunity, increasing intestinal NK-associated NKR-P1A^+^ cells and promoting maturation of lamina propria NK cells toward an adult-like phenotype, without affecting systemic antibody responses [52]. In parallel, lactoferrin, a minor whey component, enhances innate immunity by increasing NK cell activity and supporting Th1-type antiviral immune responses [53].

These data may be of particular interest in COVID-19 context for example, where NK cells have been shown to be markedly reduced and functionally exhausted, with decreased levels persisting in circulation, tissues, as well as in long COVID, and showing modulation after vaccination, including a rapid decline of NKG2A^+^ NK cells after the first dose in some studies [54,55,56,57,58].

### 2.3. Effect of Whey Intake on Innate Adaptive System

Whey protein supplementation also modulates adaptive immunity, particularly T and B lymphocyte responses. In a rat study, dietary supplementation with 1% WPC enhanced helper T cell activity after antigen exposure, indicating improved adaptive immune responsiveness [59].

Comparative studies show that whey proteins consistently enhance secondary antibody responses following antigen immunization compared with other protein sources such as soy or ovine colostral whey. Long-term feeding (5–8 weeks) also enhances cell-mediated immunity, including delayed-type hypersensitivity reactions and splenocyte proliferation, without adverse effects on general health parameters [60].

At the cellular level, whey protein products and their enzymatic digests stimulate murine splenocyte proliferation, with microfiltered whey protein isolate enhancing lymphocyte proliferation and digestion generating bioactive peptides with strong immunostimulatory activity at low concentrations [61]. In a 21-day study in 45 male mice, undenatured whey proteins (100 mg/kg) significantly increased peripheral blood mononuclear cell proliferation and doubled the chemotactic migration of B cells, T cells, and dendritic cells toward chemokines, indicating enhanced trafficking to secondary lymphoid organs [62].

Lactoferrin further contributes to adaptive immunity by promoting T cell differentiation and activation, including induction of CD4 expression in immature thymocytes and enhancement of Th2 antigen-presenting interactions [63]. It also supports B cell maturation and antigen-presenting capacity, enabling stronger humoral immune responses [63,64]. Additionally, human milk lactoferrin enhances antibody responses to T-dependent antigens up to fivefold, demonstrating potent stimulation of adaptive humoral immunity [64].

## 3. Impact on Immunoglobulin Production

Another important contribution of whey to immunity is its role in immunoglobulin production. Whey proteins naturally contain 10–15% immunoglobulins, and although these proteins are digested into amino acids and bioactive peptides, they provide an optimal pool and balance of amino acids required for the synthesis of new antibodies. This is particularly relevant for immunoglobulin A (IgA), abundant in mucosal surfaces such as the respiratory tract and critical for defense against coronaviruses and influenza, as well as immunoglobulin G (IgG), the most prevalent antibody in circulation, essential for protection against both viral and bacterial infections. Studies have shown that WP supplementation significantly increased immunoglobulin G levels [65,66].

Whey and other milk proteins undergo extensive proteolysis during digestion. In the acidic environment of the stomach, they are initially hydrolyzed by gastric pepsin and additional peptidases [67]. In the small intestine, pancreatic and brush border enzymes break proteins down into oligopeptides, dipeptides, tripeptides, and free amino acids. Thus, whey immunoglobulins are decomposed in peptide and AAs, which may bring to the human body an optimal ratio of AAs to synthesize new immunoglobulins.

Whey protein supplementation has been proposed as a strategy to counteract malnutrition and immunodeficiency in cancer patients undergoing chemotherapy. In a 12-week double-blind trial involving 42 patients in Thailand, supplementation with 40 g/day of WPI enriched with zinc and selenium significantly increased serum albumin (+2.9%) and immunoglobulin G (+4.8%) levels [65]. Notably, GSH levels rose by 11.7% in the intervention group, whereas they declined by 6.0% in controls. These findings indicate that WPs can improve nutritional status, immune function, and antioxidant defenses, thereby reducing the risk of malnutrition during chemotherapy. In a rat study, diets supplemented with 1% WPC, providing isocaloric and isonitrogenous conditions compared to controls, enhanced IgG against antigen by modulating helper T cells which were increased at days 14 and 21 [59].

A study in BALB/c mice examined the effect of dietary bovine WPC on gut mucosal immunity. Mice were fed a diet containing 20% WPC for 12 weeks and orally immunized with cholera toxin (CT) and ovalbumin (OVA). WPC-fed mice showed significantly higher mucosal antibody responses compared to control mice [68]. Also, strong evidence shows that beyond total protein intake, specific amino acids, especially glutamine, glutamate, and arginine, and possibly methionine, cysteine, and threonine, are crucial for optimal intestinal and GALT immune function and WPC is particularly rich in these AAs. They support epithelial integrity and growth, enhance T cell activity, promote IgA secretion, and help regulate inflammatory cytokines [69,70]. Also, oral administration of glycomacropeptide (GMP), another component of whey (up to 25%), stimulates humoral mucosal immunity through the production of antibodies at the surface of the intestinal barrier and other mucosa (secretory immunoglobulins A or SIgA), which protects it from toxins and pathogenic microorganisms [71]. These antibodies are crucial for fighting SARS-CoV-2 infection and COVID-19 efficiently for example [72] or influenza virus [73] but also in mucosal immune disorders such as inflammatory bowel disease, food allergy, celiac disease, metabolic dysbiosis, autoimmunity, and respiratory inflammation [74].

## 4. Antiviral and Antimicrobial Activities of WPs and Derivates

### 4.1. Antiviral and Antibacterial Spectrum of Whey Proteins

Milk proteins exhibit antiviral and antibacterial properties, partly through bioactive derivatives such as chemically modified proteins and enzymatically generated peptides [24,75]. When released by digestive enzymes like trypsin or pepsin, these peptides may contribute to host defense, provided they remain stable in the intestinal environment long enough to reach their target sites of action [76].

Whey proteins, including lactoferrin, GMP, α-lactalbumin, and β-lactoglobulin, exhibit broad antiviral activity against hepatitis B and C, coronaviruses, avian influenza A, human immunodeficiency virus, respiratory syncytial virus, adenovirus, cytomegalovirus, herpes simplex virus, human papillomavirus, enteroviruses, and hantavirus [24,73,77,78,79]. It also includes an activity on SARS-CoV-2 and improvements in COVID [78,80,81,82]. Table 1 summarizes how WPs, including global whey fractions as well as specific proteins and whey-derived peptides, may inhibit viral infections and decrease the severity of the related diseases.

These studies, which include in vitro, in vivo and a few clinical studies, show inhibition of viral entry and replication as well as anti-inflammatory effects and protection of several organs. But more investigations and practical use in clinical studies are needed to confirm these effects and synergies with usual treatments. Whey proteins also exhibit activity against several bacteria.

### 4.2. Antiviral and Antimicrobial Activity of Lactoferrin

Lactoferrin is a protein of particular interest and is the subject of a large number of studies. Lactoferrin inhibits viral attachment and entry by binding host cell heparan sulfate proteoglycans or viral envelope proteins, as demonstrated for hepatitis B and C viruses [24,46], human immunodeficiency virus [24], influenza virus [24,73,83], coronaviruses including SARS-CoV-2 [79,80,81,82,84,85,86], adenoviruses [24], enteroviruses [24], hantaviruses [24], cytomegalovirus [24], and herpes simplex virus [24] (Table 1). Lactoferrin also exhibits synergistic antiviral activity with classical antivirals such as acyclovir and cidofovir against herpes simplex virus and cytomegalovirus, respectively [24].

In influenza A infection, the *C*-lobe of bovine lactoferrin specifically targets the conserved HA2 fusion region of hemagglutinin, thereby blocking hemagglutination, viral fusion, cytopathic effects, and viral replication at femto- to picomolar concentrations [83]. The tetrapeptides VLRP, SLDC, and SKHS were identified as minimal fragments retaining broad anti-influenza activity.

Lactoferrin exhibits multiple antiviral mechanisms against SARS-CoV-2, including strong binding to the spike protein [87,88], competition with ACE2 for viral docking [89], and interference with the furin cleavage site [90]. It further impedes viral entry by interacting with heparan sulfate proteoglycans [91] and sialic acid residues [89], while also suppressing cathepsin L activity and modulating host immune responses [92]. In one cohort, lactoferrin-treated patients showed significantly faster viral RNA negativization than untreated patients (15 vs. 24 days; *p* < 0.001) [81]. Its antiviral activity was linked to spike protein binding, inhibition of viral entry, restoration of iron homeostasis, and anti-inflammatory effects [81].

Against SARS-CoV-2, lactoferrin enhances NK cell and macrophage activity, promotes Th1 immune responses, reduces IL-6 and TNF-α production, sequesters iron required for viral growth, and accelerates viral RNA clearance in patients [53,79,80,81,82]. In hepatitis infections, lactoferrin decreases viral load, lowers ALT and inflammatory markers, improves neutrophil phagocytic activity, and protects against liver injury through IL-11 and BMP2 upregulation [24].

Beyond direct antiviral effects, lactoferrin reduces oxidative stress, limits leukocyte infiltration and inflammatory cytokine production, and enhances innate and adaptive immune responses [53,81]. A study administered oral liposomal lactoferrin combined with zinc and vitamin C to symptomatic COVID-19 patients for 10 days [82]. Common symptoms included fatigue, anosmia, ageusia, muscle pain, dry cough, and headache, with all 75 treated patients reportedly improving within 5 days, including 12 patients receiving lactoferrin alone [82]. Although promising, these findings remain preliminary and require confirmation through larger controlled clinical trials.

These multifunctional properties make lactoferrin a promising natural therapeutic candidate for the prevention and adjunctive treatment of viral infections.

Beyond lactoferrin, other milk and WPs display antiviral potential; notably, β-lactoglobulin-derived peptides ALMPHIR and IPAVFK can disrupt infection by inhibiting cathepsins and binding to both viral and host receptors [78,88,93].

Also, lactoferrin inhibits bacterial growth through iron sequestration, disrupts bacterial membranes, and reduces adhesion to gastric epithelial cells, suggesting a potential complementary role in the management of *H. pylori* infection [94].

### 4.3. Antiviral and Antimicrobial Activity of Glycomacropeptide

Glycomacropeptide (GMP), another peptide of growing interest and the glycosylated form of caseinomacropeptide (CMP), is a milk-derived bioactive peptide that is released from κ-casein via enzymatic digestion, either physiologically or in industry during the cheese-making process [95,96]. GMP, which is present in high amounts in high-quality sweet whey (15–25%), is rich in branched-chain amino acids (leucine, isoleucine, valine) and has a high solubility in water, in addition to its heat stability [97]. This carbohydrate-containing peptide is susceptible to being enzymatically hydrolyzed during digestion but can also survive gastric transit without hydrolysis and is absorbed intact into the bloodstream and distributed by blood vessels, or it can reach the large intestine to be fermented by microbiota [98].

Glycomacropeptide, particularly κ-casein GMP, exhibits notable antiviral and immunomodulatory properties against several viral infections [46,73]. In hepatitis B virus infection, GMP in combination with lactoferrin interferes with viral attachment and entry into host cells in vitro, suggesting a protective role during early stages of infection [24]. Against influenza A virus, κ-casein GMP inhibits viral hemagglutination and prevents viral attachment to host cell receptors, thereby reducing infection efficiency [73]. Due to its high sialic acid content, GMP can act as a decoy receptor for viral hemagglutinins and other adhesion proteins, limiting virus–host interactions [73]. Beyond direct antiviral activity, GMP also displays anti-inflammatory and immunoregulatory effects, including modulation of cytokine production and support of mucosal immunity, which may contribute to host defense against respiratory and enteric pathogens [95]. Collectively, these findings identify GMP as a promising bioactive milk-derived peptide with potential applications in antiviral nutrition and adjunctive therapeutic strategies [24,46,73].

In vitro and in vivo studies have shown that GMP can bind to a range of pathogenic bacteria and toxins, including *Vibrio cholerae* cholera toxin (CT), enterohaemorrhagic *Escherichia coli* (EHEC O157) [99], as well as *Salmonella enteritidis*, *Morganella morganii*, *Salmonella typhimurium*, and *Shigella flexneri* [75,100,101]. In contrast, no binding has been observed with the probiotic bacterium *Lactobacillus casei* [75].

GMP has also demonstrated activity against oral pathogens involved in dental plaque formation and caries, such as *Streptococcus mutans*, *Streptococcus sanguis*, *Actinomyces viscosus*, and *Streptococcus sobrinus* [102,103].

Furthermore, GMP has been shown to influence the skin microbiota by preventing colonization by *Staphylococcus aureus*, which is associated with atopic dermatitis, while also supporting the growth of beneficial gut bacteria such as *Bifidobacterium* spp. [104].

In addition, studies have reported a prebiotic effect of GMP, characterized by increased growth and modulation of gut microbiota, including *Bifidobacterium infantis*, *Bifidobacterium breve*, *Bifidobacterium bifidum*, and *Bifidobacterium lactis* [105,106,107,108]. This activity is partly attributed to the sugar composition of GMP, which is particularly rich in sialic acids [109].

In light of the existing evidence, additional clinical research is needed to thoroughly evaluate the effects of whey proteins on immune enhancement and infection control.

## 5. Regulation of Gut Health and Microbiota

Another mechanism by which WPs, in particular through certain amino acids (AAs) such as glutamine, support immune function, is through the maintenance of gut health. They help preserve intestinal barrier integrity and promote a balanced gut microbiota, both of which are essential for optimal immune regulation.

### 5.1. Role of WPs in the Maintenance of a Healthy Gut Microbiota

Studies demonstrate that the gastrointestinal microbiota plays a critical role in systemic immune adaptation, influencing distant organs such as the lungs [110], which are affected in coronavirus infections, as well as the kidneys and cardiovascular system. This positions the gut as a central hub in inter-organ immune crosstalk. Also, emerging evidence indicates that alterations in the gut microbiome may contribute significantly to the pathogenesis of autoimmune diseases [111].

The gut microbiota, that some researchers have also called "the forgotten organ", is the most abundant microbial community in the body, weighing 1–2 kg and comprising approximately 10^14^ bacteria, roughly equivalent to the total number of human cells [112]. Notably, around 70% of the body’s immune system resides in the gut, reflecting its essential role in preventing pathogen colonization and systemic invasion.

The "Western diet", in particular a low-fiber, high-fat/high-carbohydrate and a diet rich in sugar like glucose as well as fructose is one factor that can lead to severe dysbiosis and gut inflammation [113,114,115]. On the contrary, amino acids (AAs) play a critical role in maintaining gut microbiota homeostasis, the balanced composition of beneficial and potentially pathogenic bacteria, fungi, and other microorganisms, which is essential for optimal immune function and overall health [116].

Current evidence from in vitro, animal, and human studies suggests that WPs can modulate gut microbiota composition and intestinal function.

In a study of 16 older adults, daily supplementation with 59 g of WPI (35 g protein) for 3 weeks significantly increased gut microbial diversity, particularly in individuals with low baseline diversity, with effects observed from day 3. WPI promoted beneficial taxa including *Ruminococcaceae*, *Faecalibacterium*, *Christensenella*, *Lactobacillus*, and *Lactococcus*, while reducing potentially harmful groups such as *Proteobacteria* [117].

In vitro fermentation models (gastrointestinal digestion following fecal batch culture fermentation, mimicking colonic fermentation, and simulator of the human intestinal microbial ecosystem—SHIME) show that whey substrates promote beneficial genera such as *Bifidobacterium* and *Lactobacillus* and support short-chain fatty acid production and decreased Bacteroides fragilis and in sulphite-reducing clostridia, especially Clostridium perfringens [118,119]. Animal studies further indicate that whey supplementation can reshape microbiota profiles and improve intestinal barrier integrity, even under pathological or high-fat diet conditions [120,121]. Additionally, WPI supplementation decreased high-fat diet (HFD)-induced intestinal permeability disruption in the distal ileum; an effect that was reversed by chronic antibiotic cocktail treatment [120]. In summary, WPI reverses the effects of HFD on metabolic and physiological functions through mainly microbiota-independent mechanisms. Moreover, they demonstrated a protective effect of WPI on HFD-induced inflammation (decrease in the expression of genes encoding pro-inflammatory markers—MCP-1, TNFα, and CD68) and ileal permeability disruption, with the latter being reversed by gut microbiota depletion. Additionally, in a valproic-acid-induced autism model, Wistar rats supplemented with WPs (1.24 mg/g body weight for 24 days) showed increased gut bacterial diversity, with a predominance of Firmicutes in supplemented groups (*p* < 0.05). Whey proteins also improved memory performance in the Y-maze and reduced solitary behavior in male rats [121].

Systematic reviews confirm that protein source and processing influence microbial outcomes, with dairy proteins exerting distinct modulatory effects [122,123]. Emerging research also highlights synergistic effects between WPs and bioactive compounds or polysaccharides as prebiotics in regulating microbial diversity and inflammatory pathways including in infants [124,125].

Collectively, these findings support a role for WPs and derived peptides in promoting gut microbiota balance, enhancing intestinal barrier function, and modulating immune–metabolic homeostasis.

Approximately 50–70% of the body’s lymphocytes and the majority (around 70–80%) of antibody-producing plasma cells are located in the gut-associated lymphoid tissue (GALT) [126]. Consequently, impairment of gastrointestinal integrity may have important effects on immune homeostasis.

### 5.2. Role of WPs in the Maintenance of a Healthy Epithelial Barrier

A healthy intestinal barrier relies on tight junctions between epithelial cells to prevent toxins, allergens, and pathogens from entering the circulation. If the intestine becomes permeable, a condition referred to as “leaky gut”, these molecules and bacteria can translocate into the bloodstream, reach distant organs, and trigger inflammation, allergic reactions, and autoimmune responses [127,128]. Increased intestinal permeability has been associated with food allergy and hypersensitivity. Studies have shown that individuals with these conditions exhibit significantly greater intestinal permeability than healthy controls, and that higher permeability correlates with more severe clinical symptoms [53].

When the gastrointestinal system is damaged or inflamed, the body must allocate additional energy and resources to restore and maintain gut integrity and immune function. Over time, sustained physiological stress may impair gastrointestinal immune regulation, which can in turn contribute to broader dysregulation of systemic immune function. This dysfunction increases susceptibility to infections and is associated with conditions such as asthma, eczema, allergies, and food sensitivities, in which the immune system overreacts to specific food components and causes tissue damage.

Some studies show that AAs can really help prevent or reverse intestinal permeability and improve gut microbiome [129] even if more studies are needed. In the small bowel mucosa, glutamine, which can be synthesized from glutamic acid (13–17 g/100 g in whey), is a unique nutrient providing fuel for metabolism, regulating cell proliferation and repairing and maintaining the gut barrier functions [130,131,132,133,134]. Glutamine supplementation also protects the gut against inflammatory conditions and other stresses [135,136,137,138]. In a colitis model, mice fed glutamine had decreased expression of inflammation receptors and had less colonic T cell infiltration compared with controls thereby decreasing inflammatory mediators in treated individuals [139]. Furthermore, depleted levels of glutamine have been associated with an impaired stress response in human lymphocytes in vitro [140].

Furthermore, other AAs like arginine, glycine, lysine, threonine, and sulfur-containing amino acids have shown to decrease gut inflammation stage and gut-related disease [134,139,141]. Arginine has been shown in vitro to improve barrier function in colon cells injured with a commonly used immunomodulatory medication (methotrexate) [142]. In several animal models of intestinal disease, intestinal permeability was maintained by arginine administration [143]. Oral arginine supplementation decreased intestinal mucosal injury following lipopolysaccharide treatment in rats [144].

Glutathione (GSH), which consists of glycine, glutamine, and cysteine, is the major intracellular low-molecular-weight thiol and plays important roles in antioxidant defense, nutrient metabolism and cytoprotective events [145]. GSH in the gut lumen and gut cells (enterocytes) is of critical importance in maintaining normal intestinal function, in part, by protecting epithelial cells from damage by oxidants and fatty acid hydroperoxides [146]. The major end products of Met and Cys metabolism are GSH, homocysteine (Hcy), and taurine (Tau), which play important roles in the intestinal immune response [147,148]. Thus, administration of specific dietary substrates and precursors for GSH synthesis like whey or *N*-acetylcysteine (NAC) is an effective strategy to improve gut mucosal functions and may prevent or treat intestinal diseases [134,144].

The interest in whey for improving epithelial barrier dysfunction has already been shown by some studies [149,150] and it decreased inflammation [151] even in patients with Crohn disease where the consumption of 0.5 g/kg of body weight/day of glutamine or for 2 months improved intestinal permeability (assessed by the lactulose mannitol excretion ratio in urine) and morphology improved significantly in both groups [141].

Interestingly, GMP intake stimulates humoral mucosal immunity through the production of antibodies at the surface of the intestinal barrier and other mucosa (secretory immunoglobulins A or SIgA), which protects it from toxins and pathogenic microorganisms [71].

Furthermore, as already mentioned, studies have demonstrated that GMP exhibits prebiotic activity by promoting the growth and modulating the composition of gut microbiota in both animals and humans, including strains such as *Bifidobacterium infantis*, *B. breve*, *B. bifidum*, and *B. lactis* [105,106,107,108]. This prebiotic effect is largely attributed to the sugar moieties in GMP, which are particularly rich in sialic acids [109].

## 6. Antioxidant/Anti-Inflammatory Activity of Whey Proteins

### 6.1. Key Role of Sulfur Amino Acids in Maintaining Redox Balance and Antioxidant Capacity

Thanks to the exceptional richness in the sulfur AAs cysteine and methionine when compared to other sources of proteins (like vegetal proteins for ex) and bioavailability, whey protein intake may enhance the synthesis of glutathione (GSH), the body’s primary intracellular antioxidant. By increasing GSH availability, it can help mitigate oxidative stress and inflammation, thereby improving overall antioxidant defenses and inflammatory status [41,152], which has immuno-enhancing effects [60].

Again, intracellular redox balance is primarily controlled by GSH and thioredoxin (Trx) systems [41], while extracellular redox potential (Eh) is largely governed by the cysteine/cystine (Cys/CySS) couple, the main low-molecular-weight thiol/disulfide buffer in plasma. Additionally, evidence suggests that WPC may selectively target tumor cells, characterized by elevated intracellular GSH, thereby enhancing their sensitivity to chemotherapy [42,153].

Thus, an improvement in GSH, which regulates oxidative state in all cells of the organism [41], can also improve the efficiency of all immune cells. The intake of NAC for example, a cysteine donor, has been shown to prevent replication of viruses like influenza and other respiratory viruses [154] and potentially attenuate symptoms and improve survival [155,156,157,158]. Furthermore, as prevention, GSH increase can also reduce susceptibility to influenza or coronavirus infection before we get exposed to viruses [159,160,161].

### 6.2. COVID-19 and the Interest of Whey in Modulating Inflammation and Oxidative Stress for Immune Balance

During viral infections such as SARS-CoV-2, severe disease has been associated with oxidative stress and inflammatory dysregulation involving the renin–angiotensin, bradykinin, and complement pathways [13,162]. Oxidative stress and inflammation are closely interconnected, as excess reactive oxygen species (ROS) may promote pro-inflammatory signaling and cellular damage, while inflammation further enhances ROS production [163]. In COVID-19, several oxidative stress biomarkers have been associated with disease severity and ICU admission [164,165,166,167], and spike-protein-induced inflammation has been linked to oxidative stress via these pathways [166]. More generally, in COVID-19 as in many infectious diseases, infection acts as the trigger, whereas immune–inflammatory dysregulation contributes to disease progression, severity, and death.

In this context, whey protein (WP) supplementation has been proposed as a supportive nutritional strategy. In ICU settings, adequate WP intake may help meet protein requirements more rapidly and potentially reduce mechanical ventilation duration, improve inflammatory markers, and support survival outcomes [168,169]. Supporting this, in 22 cystic fibrosis patients with lung inflammation, whey protein isolate (WPI) supplementation increased lymphocyte GSH by +46.6% from baseline (*p* < 0.05) after 10 g twice daily vs. casein over 3 months [170], a pathway also involving NF-κB signaling as in COVID-19 [171]. In 83 hemodialysis patients, a fermented whey beverage improved nutritional and inflammatory status, reducing subjective global assessment (SGA: −3.22 vs. +1.56, *p* < 0.001) and malnutrition–inflammation score (MIS: −1.83 vs. +1.48, *p* < 0.001) [172].

More broadly, additional evidence from intervention studies suggests immunomodulatory and antioxidant effects of WP: in an 8-week trial (*n* = 80), fermented Maillard-reactive whey proteins (F-MRP, 6 g/day with *Lactobacillus plantarum* LC01) increased IL-12 (*p* < 0.001), improved pre-albumin, and enhanced NK cell cytotoxicity at multiple effector-to-target ratios (10:1, 5:1, 2.5:1, 1.25:1; *p* < 0.001), while placebo showed reduced activity at 5:1 [60]. In mice, WP supplementation reduced IL-1α, IL-1β, IL-10, TNF-α, ROS, cholesterol, and triglycerides, while increasing IL-2, IL-4, IL-7, IL-8, and GSH (100 mg/kg body weight) [34]. A systematic review and meta-analysis of 31 RCTs further reported that whey protein reduced IL-6 (MD: −0.79; −0.98 in sarcopenia/pre-frailty) and soy protein reduced TNF-α (MD: −0.16), with effects influenced by population and intervention duration [173].

### 6.3. Sarcopenia and Immuno-Inflammation in COVID-19: Emerging Interest of Whey Proteins

Supplementation may be particularly beneficial for sarcopenic individuals. Geriatric syndromes such as sarcopenia and frailty are especially relevant given the higher COVID-19 mortality observed in older adults. During the pandemic, lockdowns, reduced physical activity, altered dietary habits, stress, sleep disturbances, and prolonged hospitalization likely contributed to muscle loss, functional decline, and higher mortality [174,175,176]. Importantly, sarcopenia directly impacts immunity and induces immunosenescence: it is associated with chronic low-grade inflammation, impaired proliferation of peripheral mononuclear cells, and altered monocyte activity, all of which compromise the body’s defense against infections [177,178].

More broadly, preventive strategies targeting sarcopenia, including whey intake, vitamin D supplementation, and exercise, may therefore contribute to healthier aging and improved immune resilience [179,180,181].

### 6.4. Nitro-Oxidative Stress and Immune Regulation: Modulatory Effects of Whey Proteins

The immune–inflammatory response is closely linked to nitro-oxidative stress, where ROS/RNS regulate immune cell activation, metabolism, and function via redox-sensitive pathways including NF-κB, HIF-1α, mTOR, PI3K/Akt, MAPKs, AMPK, and PPAR [182]. Antioxidant systems such as glutathione, thioredoxin, Nrf2, and the HDL/ApoA1/PON1 complex maintain redox balance and regulate macrophage and T cell polarization, cytokine production, phagocytosis, and immune tolerance. In contrast, chronic nitro-oxidative stress and hypernitrosylation impair antioxidant defenses, mitochondrial function, and immune metabolism, leading to immune dysregulation.

Clinically, whey protein (WP) supplementation (20 g/day for 3 weeks) in 42 ischemic stroke patients (19 WP vs. 21 control receiving routine formula) significantly reduced NIHSS and mRS scores, as well as TNF-α, IL-6, and hs-CRP (*p* < 0.05) [183]. In experimental models, WPs attenuated oxidative-stress-induced skeletal muscle injury and improved recovery after exercise-induced damage, enhancing cell migration, differentiation, muscle morphology, function, and protein synthesis [184]. These effects involved activation of the SIRT1/Nrf2/HO-1 axis and downstream AMPK/TSC2/mTOR/4EBP1 signaling.

### 6.5. GMP in Immunity, Metabolism, and Mineral Balance

The anti-inflammatory and immunomodulatory effects of GMP have been reported in animal models of inflammatory bowel disease and in various while blood cells (dendritic cells, lymphocytes, and macrophages) [185,186,187,188,189,190]. Furthermore, GMP may have an interest in diabetes through the management of fasting blood glucose levels, insulin production, and insulin resistance [71,185,191] and may contribute to a significant part of the anti-diabetes activity of whey, which we have reviewed recently [26,192].

Of note, GMP has in vivo remineralizing properties in teeth [193] and more generally in the body, such as on zinc corporal absorption [194], which is a gatekeeper of immunity [195], and on calcium incorporation and bone strength [196,197].

### 6.6. Bovine Lactoferrin and Its Anti-Inflammatory Potential

Bovine lactoferrin from milk, a protein present in whey, acts as a natural immunomodulator. In a rat model of rheumatoid arthritis, oral BLF (100 mg/kg) reduced arthritis development, hyperalgesia, and pain, both preventively and therapeutically [198]. BLF suppressed pro-inflammatory TNF-alpha and increased anti-inflammatory IL-10 production. These findings suggest BLF’s potential as a safe natural treatment for rheumatoid arthritis, targeting both inflammation and joint pain.

Lactoferrin was tested in murine models of collagen-induced and *S. aureus* septic arthritis by periarticular injection (0.5–1 mg). It markedly reduced local inflammation for up to 3 days, reaching ~71% of corticosteroid efficacy, while ~25% of injected protein persisted locally after 6 h [199]. Serum IL-6 levels were unaffected, indicating a local rather than systemic effect. Importantly, in septic arthritis, lactoferrin suppressed inflammation without promoting bacterial survival, supporting its therapeutic value.

### 6.7. Lysine and Arginine in Oxidative Stress and Immune Regulation

Furthermore, lysine and arginine, present in high concentrations in WPs, contribute to non-enzymatic antioxidant defenses by scavenging free radicals and modulating oxidative reactions [200]. Arginine has been shown to attenuate inflammation and oxidative stress by activating arginase-1 signaling and reducing LPS-induced ROS and pro-inflammatory mediators [201]. Through regulation of nitric oxide synthase (NOS) and arginase pathways, arginine critically modulates immune and inflammatory responses [202]. Clinically, the combination of lysine and arginine has been associated with improved neutrophil migration, chemotaxis, lymphocyte cytotoxicity, and increased IgG levels in patients with recurrent infections [203]. Together, these findings support a synergistic antioxidant, anti-inflammatory, and immunomodulatory role for lysine and arginine across cellular and clinical contexts.

The interplay between oxidative stress, inflammation, and immune regulation is highly complex, involving tightly interconnected redox-sensitive signaling pathways that shape immune cell function and metabolic responses. Current evidence suggests that whey proteins may help modulate this redox–inflammatory–immune axis, but further mechanistic and well-designed clinical studies are still needed to confirm and refine these effects.

**Figure 1:**
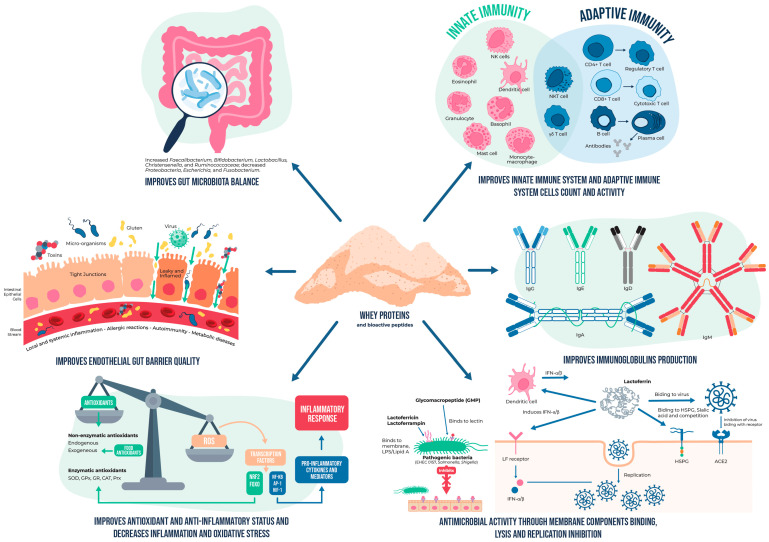
Mechanisms underlying whey proteins (WPs)–mediated immune enhancement and infection resistance.

Figure 1 summarizes how WPs, including global whey fractions as well as specific proteins and whey-derived peptides, support immune function regarding the elements discussed in this review. These bioactive components enhance innate and adaptive immunity, exert direct antiviral and antibacterial effects, and modulate host defenses through immunoregulatory, antioxidant, and anti-inflammatory mechanisms, while also helping maintain intestinal homeostasis and microbiota balance, which are important for infection prevention, immune boosting, and responses during infection.

In addition, Table 1 summarizes the potential of whey proteins, including whole whey fractions as well as specific proteins and bioactive peptides, to inhibit viral infections and reduce disease severity.

**Table 1:** Inhibition of viral infections by whey proteins and modes of action.

Target	Whey Proteins	Mode of Action	References
Bloodborne/chronic infections			
Hepatitis B virus	Lactoferrin and GMP	Increases hepatic GSH, IL-2, and NK cell activity (not in hepatitis C) in men	[46]
	Lactoferrin	Reduces inflammation (AST and ALT) and protects against hepatitis liver damage (increases IL-11 and BMP2) in mice	[151]
	WPs	Interferes with viral attachment and entry in vitro	[24]
Hepatitis C virus	WPs	Decreases viral load, normalizes serum albumin, and improves phagocytic function of neutrophils in humans (decrease in serum levels of ALT, ICAM-1, IL-2, and NO).	[204]
	Lactoferrin	Decreases viral load in humans (and ALT)	[205]
Human immunodeficiency virus	Chemically altered casein, β-lactoglobulin, and α-lactalbumin	HIV-1 replication (binds to HIV-1 gp 120 envelope glycoprotein)	[206]
	Modified α-lactalbumin	Inhibits HIV-1 replication in vitro	[207]
	WPs	Improves oxidative stress (glutathione restoration) and support muscle mass and nutrition	[208]
Respiratory viruses			
Coronaviruses, SARS-CoV-2	Lactoferrin	Increases NK cell activity and supports Th1-type response	[53]
	Lactoferrin	Blocks viral entry (binding heparan sulfate), enhances NK cell and macrophage activity, promotes Th1 immune balance in vitro, reduces inflammatory cytokines (L-6, TNF-α) in animal models, sequesters iron	[79,80,82]
	Lactoferrin	Fasters SARS-CoV-2 RNA negativization (about 15 vs. 24 days), symptom reduction especially in older patients	[81]
	Lactoferrin	Rapid symptom improvement within ~5 days in a small subgroup (*n* = 12 subjects)	[82]
	WPs	Prevents sarcopenia and frailty in seniors and elderly which are associated with higher mortality	[174]
Influenza A virus	K-casein GMP and lactoferrin	Inhibits virus hemagglutination, blocking viral attachment to host cells	[73]
	Modified β-lactoglobulin	Inhibits influenza A and B by binding hemagglutinin (HA1) and blocking viral attachment to sialic acid receptors; intranasal administration protects mice from influenza infection in vivo	[77]
	Lactoferrin (especially its *C*-lobe)	Binds influenza A hemagglutinin, inhibitis fusion peptide activity and viral entry in vitro	[83]
	Methylated β-lactoglobulin	Reduces lung inflammation, leukocyte infiltration, and IL-6 levels in influenza A(H1N1) in mice Reduces viral RNA replication	[209]
	Lactoferrin	Binds the HA2 region, inhibiting hemagglutination, cytopathic effects, and viral infection	[210]
	Lactoferrin (particularly its *C*-lobe)	Inhibit influenza virus infection by targeting the conserved HA2 fusion region of hemagglutinin and blocking multiple stages of viral entry and cytopathic effects	[211,212]
Respiratory syncytial virus	Lactoferrin	Reduces tachypnea and weight loss in mice (intranasal); anti-inflammatory and immunomodulatory	[213]
Adenovirus	Lactoferrin	Inhibits viral replication throughout the viral cycle by binding viral particles and targeting structural polypeptides III and IIIa	[214,215]
DNA viruses with latency/persistence			
Cytomegalovirus	Methylated alpha-lactalbumin	Interferes with viral genomic DNA, disrupting replication and transcription processes	[216]
	Lactoferrin	Synergistic antiviral activity with cidofovir against in vitro by inhibiting viral entry and DNA synthesis	[217]
Herpes simplex virus	Chemically modified bovine serum albumin, α-lactalbumin, β-lactoglobulin,	Shows in vitro anti-HSV-1 activity at multiple stages of infection	[77]
	Lactoferrin	Binds strongly to heparan sulfatel; early infection steps and shows synergistic activity with acyclovir	[218,219]
Human papillomavirus	Chemically modified bovine β-lactoglobulin	Inhibits viral entry and early stage of viral replication	[220,221]
Enteric/systemic viruses			
Enteroviruses	Lactoferrin	Prevents viral attachment by blocking adsorption or receptor-mediated binding to the host cell membrane	[222]
	Lactoferrin	Partially inhibits Enterovirus E replication (1–1.1 log) and reduced intracellular viral RNA (75%)	[223]
Emerging/zoonotic viruses			
Hantavirus	Lactoferrin	Blocks viral attachment to host cells and reduces the shedding of virus particles in vitro and in mice	[224,225]

Importantly, the antioxidant and anti-inflammatory properties of WPs strongly depend on their processing quality. Low-temperature, non-acidic microfiltration preserves the native structure of bioactive proteins and protects sensitive amino acids, particularly sulfur amino acids and tryptophan, essential for glutathione synthesis and redox regulation [26]. In contrast, excessive heat or harsh pH treatments can denature proteins, promote aggregation and oxidation, and reduce their biological activity, thereby compromising their antioxidant and immunomodulatory potential [29,30,31].

## 7. Antimicrobial Modes of Action of Whey-Protein-Derived Peptides: Bioavailability, Structure–Activity, and Cellular Interactions

### 7.1. Bioavailability and Generation of Active Peptides

Whey-protein-derived peptides, particularly lactoferrin fragments, are important bioactive molecules with antibacterial and antiviral activities. Lactoferricin, lactoferrampin, and LF1-11 (*N*-terminal peptide of lactoferrin) are generated through pepsin-mediated proteolysis of lactoferrin during gastric digestion, supporting their physiological bioavailability in vivo [226,227,228]. Lactoferricin is a cationic *N*-terminal peptide 17–41 containing a disulfide-bonded loop (FKCRRWQWRMKKLGAPSITCVRRAF), whereas lactoferrampin derives from residues 265–284 of the lactoferrin N1-domain (WKLLSKAQEKFGKNKSR) and adopts an amphipathic α-helical structure [227,229,230].

Comparable fragments are also released from α-lactalbumin (GYGGVSLPEWVCTTF ALCSEK (residues (17–31)S-S(109–114)) and β-lactoglobulin (VAGTWY (residues 15–20), AASDISLLDAQSAPLR (residues 25–40), IPAVFK (residues 78–83) and VLVLDTDYK (residues 92–100)) after digestion by pepsin, trypsin, or chymotrypsin, and have been proposed to be responsible for antimicrobial activity [231,232].

### 7.2. Structure–Activity Relationships

The antimicrobial activity of WP-derived peptides is mainly determined through cationicity, amphipathicity, hydrophobicity, and conformational flexibility [228,230]. Lactoferricin contains positively charged Arg and Lys residues together with hydrophobic Trp and Phe residues that promote membrane binding and destabilization [227,233]. Lactoferrampin possesses a positively charged *C*-terminal region essential for electrostatic attraction and an *N*-terminal amphipathic α-helix stabilized by aromatic residues including Trp1 and Phe11 [229,230]. NMR and membrane studies demonstrated preferential interaction with negatively charged phospholipids and membrane-active conformations after lipid binding [234].

### 7.3. Cellular and Membrane-Level Interactions

Whey-protein-derived peptides primarily act through membrane-targeting mechanisms (see Figure 1). Their cationic nature promotes electrostatic interactions with negatively charged microbial surfaces such as lipopolysaccharides (LPSs), phospholipids, and teichoic acids [228,235,236]. Amphipathic structures then insert into lipid bilayers, causing membrane permeabilization, depolarization, pore formation, and leakage of intracellular components. Lactoferricin disrupts bacterial membranes and inhibits macromolecular synthesis [237,238]. Lactoferrampin rapidly penetrates microbial membranes and induces ultrastructural damage, including vesicle formation in *Candida albicans* and membrane detachment in *Escherichia coli* [229].

This membrane-disruptive activity confers broad-spectrum efficacy against Gram-negative (*Escherichia coli*, *Pseudomonas aeruginosa*, *Salmonella* spp., *Klebsiella pneumoniae*) and Gram-positive (*Staphylococcus aureus*, *Streptococcus* spp., *Listeria monocytogenes*) bacteria, as well as *Candida albicans*, via binding to negatively charged microbial surfaces [227,228,229,235].

### 7.4. Antiviral Mechanisms

Lactoferrin-derived peptides exhibit antiviral activity against herpes simplex virus, human cytomegalovirus, hepatitis C virus, human immunodeficiency virus, and influenza virus [83,218,219,228,239]. Their principal mechanism involves inhibition of viral attachment and entry through interactions with viral envelope proteins or host cell receptors such as heparan sulfate proteoglycans. In influenza A virus, the *C*-lobe of bovine lactoferrin binds the HA2 fusion domain of hemagglutinin under acidic endosomal conditions, thereby interfering with membrane fusion [83,240]. Furthermore, minimal tetrapeptides including VLRP, SLDC, and SKHS retained potent anti-influenza activity [83].

### 7.5. Endotoxin Neutralization and Immunomodulation

Lactoferricin-derived peptides also exert anti-inflammatory effects through LPS neutralization. Binding to lipid A involves electrostatic interactions mediated by cationic residues and hydrophobic insertion driven by tryptophan residues [228,236,241]. This interaction destabilizes LPS aggregates, suppresses endotoxin activity, and reduces IL-6 production [242,243]. Structural studies further demonstrated conformations optimized for phosphate group recognition within lipid A, explaining its dual antimicrobial and anti-inflammatory properties [244].

Overall, whey-protein-derived peptides constitute a multifunctional antimicrobial system activated by proteolysis with possible action on parasites [245]. Their activities involve peptide release, electrostatic membrane recognition, amphipathic insertion, pathogen disruption, viral entry inhibition, and endotoxin neutralization. Lactoferrin-derived peptides, particularly lactoferricin and lactoferrampin, therefore represent promising templates for novel broad-spectrum antimicrobial and antiviral therapeutics.

Table 2 summarizes the current experimental and clinical evidence on the immunomodulatory efficacy of whey proteins and their bioactive peptides in vivo, together with the mechanisms underlying these effects. The table compiles findings from in vitro, in vivo, and clinical studies, highlighting the ability of WPs and derived peptides to modulate immunity.

Key outcomes include effects on innate and adaptive immune cells, impact on immunoglobulin production, antiviral and antimicrobial activities, regulation of gut health and microbiota, and antioxidant and anti-inflammatory activity underlying immunomodulatory efficacy. Mechanisms of action include glutathione-mediated redox regulation, immune cell and cytokine modulation, direct antimicrobial/antiviral effects via membrane disruption and binding inhibition, and gut microbiota and epithelial barrier modulation leading to reduced inflammation.

## 8. Discussion

Whey proteins, the most abundant proteins in human milk (60–80%), support innate mucosal immunity in early life, including colostrum, and may have protective effects in certain immune disorders [246,247]. Their conserved structure, high essential amino acid content, and evolutionary adaptation for efficient digestion and utilization of whey and casein support their nutritional relevance beyond infancy.

Whey proteins are "fast" proteins, producing a rapid postprandial rise in plasma amino acids due to faster gastric emptying and digestion than casein and other proteins [26,248,249,250], and are highly efficient for stimulating muscle protein synthesis.

Animal whey proteins (WPs) constitute a bioactive milk fraction with marked molecular heterogeneity and structural flexibility that underlies their immunomodulatory activity. Major components (β-lactoglobulin, α-lactalbumin, glycomacropeptide, immunoglobulins, lactoferrin) contain structural motifs controlling stability, receptor interaction, and proteolysis, leading to bioactive peptide release and regulation of immune functions (redox balance, cytokine signaling, antimicrobial defense). Detailed characterization of composition and processing-induced structural changes is required to clarify their roles in innate and adaptive immunity and in protection against viral and bacterial infections.

As described in this review, WPs can modulate the immune system through multiple pathways, which collectively may provide significant benefits to overall immune health. We have categorized these benefits through reinforcement of immune cell production and efficiency, regulation of gut health and microbiota, and antioxidant/anti-inflammatory activity.

First, various in vitro and in vivo studies suggest that WPCs enhance innate and adaptive immunity by increasing lymphocyte proliferation, chemotaxis, and neutrophil function [34,35,36] and support T and B cell maturation and migration [62,63]. Cysteine and methionine support glutathione synthesis in all immune cells, supporting the regulation of NK and T cell activity for example [40,51]. Also, animal studies show enhanced mucosal NK and helper T cell responses [56,57].

In parallel, WPs support immunoglobulin synthesis by providing an amino acid profile that promotes IgA at mucosal surfaces and circulating IgG despite being digested into peptides and amino acids [67]. Whey increases serum IgG and antioxidant status in immunocompromised patients [65,66], while animal studies show enhanced antigen-specific IgG and mucosal antibody responses [57,68]. Rich in glutamine, arginine, and cysteine, WPs support gut-associated lymphoid tissue and IgA secretion [69,70], and GMP further stimulates secretory IgA, reinforcing mucosal antiviral defense [71,72].

Furthermore, WPs support immune function via intestinal barrier integrity and gut microbiota modulation, key in systemic immunity [110,112]. WPI increases microbial diversity, promotes *Ruminococcaceae*, *Faecalibacterium*, *Lactobacillus*, and *Bifidobacterium*, enhances short-chain fatty acids, and reduces pathobionts [118,251]. Glutamine and cysteine strengthen tight junctions, reduce permeability, and enhance glutathione antioxidant defense [130,144] and supplementation attenuates inflammation and barrier dysfunction in colitis and high-fat diet models via pro-inflammatory mediator modulation [120,139], helping prevent intestinal permeability [141,149]. GMP shows prebiotic and mucosal immunostimulatory effects, promoting beneficial microbiota and secretory IgA [109].

Milk and whey proteins (WPs) exert antibacterial and antiviral effects through native proteins and bioactive peptides [24,74]. Lactoferrin, α-lactalbumin, β-lactoglobulin, and GMP inhibit viral attachment and replication against hepatitis B and C, coronaviruses and COVID-19, avian influenza A, human immunodeficiency virus, respiratory syncytial virus, adenovirus, cytomegalovirus, herpes simplex virus, human papillomavirus, enteroviruses, and hantavirus as summarized in Table 1 [23,24,62,77].

In the COVID context, lactoferrin, which binds viral proteins, has been suggested to compete with ACE2, regulates iron homeostasis, enhances NK/Th1 responses, and induces IFN-α and dendritic cells [81,82,89,252]. Meta-analysis data suggest lactoferrin (≈100–1000 mg/day) may reduce COVID-19-related fatigue, although evidence remains limited and heterogeneous [253,254].

Glycomacropeptide also shows antimicrobial, anti-adhesive, and prebiotic effects, promoting beneficial microbiota such as *Bifidobacterium* spp. [75,105,109]. Overall, WPs are multifunctional bioactive components with potential roles in infection control requiring further clinical investigation.

Finally, effective immunity depends on a balance between inflammatory and oxidative responses, where physiological ROS support antimicrobial defense, while chronic oxidative stress drives immune dysfunction and tissue damage. Nutrition contributes to immune and antioxidant regulation via vitamins, trace elements, omega-3 fatty acids, and cytoprotective pathways [255,256,257,258]. COVID-19 severity has been linked to impaired baseline redox and immune status with dysregulated immuno-inflammatory responses [13]. During the COVID-19 pandemic, public health strategies primarily focused on pharmaceutical interventions and vaccination, while comparatively less attention was given to approaches aimed at supporting baseline immune function and oxidative balance in high-risk populations [21,259]. This review highlights the potential of functional foods as complementary approaches to conventional therapies for preventing and managing infectious and immune-related diseases, which implies the need for further research, particularly well-designed clinical studies.

Whey proteins (WPs) exert antioxidant and anti-inflammatory effects mainly via cysteine- and methionine-driven glutathione synthesis and redox regulation [26,41,58,152]. Evidence shows reduced inflammatory and oxidative markers with improved GSH and NK activity in conditions such as COVID-19, stroke, cystic fibrosis, and hemodialysis [164,166,170,173,183]. Lactoferrin and glycomacropeptide further provide immunomodulatory effects in inflammatory and metabolic disorders [26,185,198], while preservation of native whey structure is important for maintaining bioactivity [26,29].

Accordingly, WPs also show beneficial effects in cardiovascular, intestinal, rheumatic, and age-related inflammatory conditions [141,149,150,151,260,261,262,263,264,265]. Overall, reviews suggest dairy, particularly WPs, generally exerts beneficial or neutral effects on inflammatory biomarkers and immune pathways [266,267,268,269,270]. Bioactive components of milk can modulate immune pathways such as Toll-like receptor signaling, influencing inflammatory responses [263]. Experimental studies additionally indicate potential neuroprotective and anticancer effects, although human evidence remains limited [271,272,273,274,275,276].

Overall, the evidence points toward potential anti-inflammatory effects, although further well-designed studies with standardized protocols are still needed.

In the future, mechanistic and clinical research should particularly focus on short bioactive peptides contained in WPs. Indeed, whey-protein-derived peptides, mainly generated by gastrointestinal proteolysis of lactoferrin, α-lactalbumin, and β-lactoglobulin, include bioactive fragments such as lactoferricin, lactoferrampin, and related peptides with demonstrated antimicrobial and antiviral potential [226,227,228].

Their activity is largely driven by structural features including cationicity, amphipathicity, and hydrophobic residues, which enable strong interactions with microbial membranes [228,230,233]. These peptides bind negatively charged bacterial surfaces (e.g., LPS and phospholipids), leading to membrane disruption, pore formation, and intracellular leakage [228,235]. Lactoferricin additionally inhibits macromolecular synthesis, while lactoferrampin induces membrane deformation and ultrastructural damage in bacteria and fungi [229,238]. Beyond antibacterial effects, they also block viral attachment and entry by targeting viral envelope proteins or host receptors such as heparan sulfate proteoglycans [239,240]. Lactoferrin-derived peptides further neutralize endotoxins by binding lipid A and suppressing pro-inflammatory cytokine production such as IL-6 [236,242].

Overall, these peptides act through combined membrane-targeting, antiviral entry inhibition, and immunomodulatory mechanisms, conferring broad-spectrum antimicrobial activity which could be key in the explanation of WPs’ efficiency in vivo [227,228].

Of note, immune imbalance and deficiency are particularly relevant in relation with aging and immune aging, also referred to as immunosenescence, and characterized by structural and functional alterations in immune organs affecting both innate and adaptive immunity, which may significantly contribute to the development of sarcopenia [277,278].

WPs and their amino acids efficiently support immune cell synthesis and function, which is particularly relevant in aging and immunosenescence, a decline in innate and adaptive immunity contributing to sarcopenia [277,278]. Current recommendations for older adults suggest ~0.4 g protein/kg per meal or up to 1.2–1.6 g/kg/day [279]. However, aging-related mastication impairment can reduce protein absorption and postprandial protein synthesis, as shown in denture wearers consuming beef [280].

The 2025–2030 Dietary Guidelines for Americans, recently released by the U.S. Departments of Agriculture (USDA) and Health and Human Services (HHS), recommend a daily protein intake of 1.2–1.6 g/kg body weight, an increase from the previous 0.8 g/kg minimum, representing a meaningful advancement, particularly for older adults [281]. These recommendations therefore emphasize the importance of high-quality, digestible protein sources to preserve muscle mass and metabolic health during aging.

In contrast, many plant proteins show lower digestibility because of anti-nutritional compounds such as phytates, lectins, saponins, and tannins, which impair protein and mineral absorption and may contribute to essential amino acid deficiencies, particularly in vegans [282,283,284,285,286,287,288,289]. Consequently, WPs appear to be a valuable protein source for supporting healthy aging, including prevention of immunosenescence and sarcopenia [253].

To conclude, and importantly, the best WPCs and WPIs are obtained with high-quality milk from grass-fed cows processed by ultra- and microfiltration at low temperature (cold-process), without applying extreme pH (acid or basic), as is it used in "ion exchange" processes, in order to protect the most sensitive amino acids (AAs) such as sulfur amino acids (sAAs), lysine, arginine, and others. However, most of the whey encountered on the market are processed with heat and/or with strong acids or bases (in presence of solvents), both of which can denature the 3D structure of proteins and may oxidize some key AAs that have strong health benefits for humans [29,30,31].

These AAs in particular, as well as several biopeptides, can contribute efficiently to boosting the immune system and help the body fight against pathogen infections like influenza, coronaviruses, as well as bacteria through different pathways [32,33]. This may be relevant for the general population, and particularly for athletes, in whom strenuous or prolonged exercise can induce transient immune alterations and increase the risk of upper respiratory tract infections (URTIs) [290].

Given their rich and diverse bioactive composition, WPs and WPCs represent promising dietary supplements for supporting immune health and overall well-being, owing to their immunomodulatory, antimicrobial, and antioxidant properties; however, further well-designed clinical studies are required to confirm these effects and fully elucidate their mechanisms of action in humans.

## Figures and Tables

**Table 2:** Experimental and clinical evidence on the immunomodulatory efficacy of WPs and peptides and their underlying mechanisms.

Section	Topic/WP Component	Study Type	Biological Effect Reported	Proposed Mechanism	Evidence Level	References
Effects of Whey Protein Intake on Innate and Adaptive Immune Cells	Whey proteins	Animal study (mice)	Increased lymphocyte count and GSH levels in liver; inhibits hydroperoxide and ROS in leukocytes and liver	Increased glutathione (GSH) synthesis via cysteine/methionine supply	Moderate–high	[35]
	Whey proteins	Clinical study	Increased GSH in mononuclear cells	Increased glutathione (GSH) synthesis via cysteine/methionine supply	Moderate–high	[38]
	Whey proteins	In vitro/mechanistic	Enhanced neutrophil and macrophage activity	Activate human neutrophils; increasing IL-6, IL-8, and TNF-α production and enhancing neutrophil priming	Low–moderate	[40]
	Non-heated whey proteins	Clinical study (chronic hepatitis patients)	Increased hepatic GSH and NK cell activity	Immunomodulation and redox metabolic reprogramming	Moderate–high	[46]
	Fermented whey proteins	Randomized clinical trial	Increased NK cell activity and IL-12	Enhanced NK activity, antioxidant, and metabolic functions	Moderate–high	[50,51]
	WPC supplementation	Animal study (suckling Wistar rats)	Enhanced intestinal NK cells and CD8αα^+^ intraepithelial lymphocytes	Early life immunonutrition enhances mucosal innate immune maturation	Moderate	[52]
	WPC supplementation	Animal study (rats)	Increased helper T cells, IL-2/IL-4, and IgG production after antigen exposure	Adaptive immune activation through helper T cell modulation and enhanced humoral responses	Moderate	[59]
	Whey proteins	Animal study (mice)	Enhanced antibody production, lymphocyte proliferation, and chemotaxis	Immunomodulation via cytokine regulation, lymphocyte activation, and immune-cell migration	Moderate	[60,62]
	Lactoferrin	In vitro/mechanistic	Increased T cell differentiation and B cell maturation	Modulation of antigen presentation and CD4 expression	Low–moderate	[63,64]
	Whey proteins vs. soy protein and other whey sources	Animal study (mice)	Enhanced secondary humoral antibody responses; delayed-type hypersensitivity and lymphocyte proliferation	Immunoactive whey fractions (bioactive proteins/peptides) modulate systemic and cellular immune responses vs. soy protein isolate and ovine colostral WPs	Moderate	[60]
	Whey protein vs. casein	Animal study (A/J mice, dimethylhydrazine-induced colon cancer)	Enhanced humoral immunity (plaque-forming cells) and reduced tumor incidence and size vs. casein and standard chow	Enhanced immune surveillance and tumor suppression potentially linked to whey-associated immunostimulation and maintenance of systemic immune function during carcinogenic stress	Moderate	[153]
	GMP and derivates	In vitro (U937cells)	Increased macrophage proliferation and phagocytic activity	Immunomodulation mediated by sialic acid-rich carbohydrate chains	Low–moderate	[189]
	WPC	Clinical study	Reduced viral load, ALT, AST; improved neutrophil phagocytosis and albumin	Immunomodulation and enhanced host defense	Moderate	[204]
Impact on Immunoglobulin Production	Whey proteins	Clinical trial (cancer patients)	Increased serum IgG, GSH levels, and antioxidant status	Amino acid supply and GSH support	Moderate–high	[65]
	WPC supplementation	Animal study	Increased mucosal antibody responses	Enhanced gut-associated immune activation	Moderate	[68]
	GMP	Animal study (mice)	Reduced colonic inflammation; increased mucosal IgA; restored immune balance	Anti-inflammatory and mucosal IgA modulation (epithelial protection)	Moderate	[74]
Antiviral and Antimicrobial Activities of WPs and Derivates	GMP and lactoferrin	In vitro (hemagglutination inhibition assay model)	Influenza hemagglutination inhibited (reduced viral binding to red blood cells)	Blocked viral attachment via hemagglutinin binding inhibition and glycan mimicry effects	Low–moderate	[73]
	GMP and glyco-conjugates (XOS, CMD) against intestinal infection	In vitro (adhesion assay in Caco-2 cells)	Reduced *E. coli* adhesion; decreased IL-8 response (*Salmonella*) and pathogen binding	Glycan binding; reduced adhesion; improved anti-adhesive and immune effects	Low–moderate	[76]
	Chemically modified WPs (3-HP derivatives)	In vitro (HSV-1/Vero)	Inhibited HSV-1 infection; antiviral activity across pre-, co-, and post-infection conditions	Enhanced viral/host interaction interference; multi-step inhibition of HSV-1 entry/replication	Low–moderate	[77]
	3-HP β-lactoglobulin	In vitro *+* in vivo (mouse intranasal challenge) study	Inhibited multiple influenza strains; protected mice from viral challenge; stable and safe profile	Binds HA1; blocks HA–sialic acid attachment; inhibits viral entry	Moderate–high	[78]
	Lactoferrin	Retrospective clinical study	Faster viral clearance; reduced symptoms	Spike and heparan sulfate binding; reduced viral entry; anti-inflammatory iron homeostasis effects	Moderate	[81]
	Liposomal lactoferrin	Prospective observational study	Rapid recovery; reduced symptoms, possible prevention in contacts	Antiviral, immunomodulatory, anti-inflammatory effects; entry inhibition	Moderate	[82]
	α-lactalbumin with GMP	In vitro (adhesion assay in Caco-2 cell)	Reduced bacterial association with intestinal epithelial cells	Competitive inhibition of pathogen adhesion; digestion-resistant peptides retain anti-adhesive activity	Low–moderate	[100]
	GMP	In vitro/porcine cells + animal study (piglets)	Reduced *E. coli* adhesion; increased *Lactobacillus*, decreased enterobacteria	Inhibited mucosal adhesion, shifted microbiota toward beneficial bacteria	Moderate	[101]
	GMP	In vitro (salivary pellicle model *+* ex vivo dental enamel model, electron microscopy)	Reduced adhesion of *Streptococcus mutans* and *S. sobrinus*; altered oral biofilm composition	Incorporation into salivary pellicle; blocked bacterial binding sites; altered biofilm ecology	Moderate	[103]
	GMP	In vitro (human keratinocyte model)	Reduced inflammation (TSLP, IL33, TARC, MDC) and oxidative stress; improved cell survival and wound healing	Inflammatory mediator suppression, oxidative stress reduction, migration, and tissue repair promotion	Moderate	[104]
	GMP	In vitro (*Bifidobacterium* growth assay + transcriptomics)	Increased bacterial growth; altered gene expression linked to carbohydrate utilization	O-linked glycan–dependent utilization, activation of glycoside hydrolase pathways, and metabolic gene regulation	Moderate–high	[109]
Regulation of Gut Health and Microbiota	GMP	In vitro (growth assay)	Enhanced probiotic bacterial growth (*Lactobacillus rhamnosus* and *Bifidobacterium thermophilum*)	Acts as a growth-promoting substrate for probiotic bacteria independently of glycosylation	Low	[107]
	GMP	In vitro (artificial colon model, fecal fermentation)	Increased microbiota diversity; enrichment of beneficial taxa; reduced *Proteobacteria* expansion	Prebiotic fermentation effects shaping microbial composition and metabolic output	Moderate	[108]
	Whey proteins	Clinical study (older adults)	Increased diversity, icreased *Faecalibacterium*, *Ruminococcaceae*, *Christensenella*, *Lactobacillus*; decreased *Proteobacteria*, *Streptococcaceae*	Diet-driven microbiota modulation, selective fermentation favoring beneficial taxa	Moderate–high	[117]
	WPI and whey retentate	In vitro fecal batch fermentation (human donors: normal-weight & obese)	Increased *Bifidobacterium*, *Lactobacillus* (especially in obese donors) and SCFA production; altered SCFA/BCFA profiles; decreased obesity-associated bacteria	Prebiotic fermentation of whey selectively stimulates beneficial microbiota and SCFA production, suppressing pathogenic/obesity-associated taxa	Moderate	[118,119]
	WPI	Animal study (mice) with microbiota depletion (antibiotics)	Reduced ileal and adipose inflammation (MCP-1, TNFα, CD68); improved intestinal permeability (ileum)	Mainly microbiota-independent metabolic and anti-inflammatory effects, partial gut barrier protection, mixed microbiota dependence	Moderate–high	[120]
	Whey proteins	Animal study (rat ASD model)	Increased microbiota diversity and Firmicutes; improved memory and social behavior	Gut–brain axis modulation via microbiota changes, microbiota-driven neurobehavioral improvement	Moderate	[121]
	Whey and glutamine	Clinical study (patients with Crohn’s disease)	Improved intestinal permeability and villous morphology from baseline; whey as efficient as glutamine	Supports enterocyte metabolism, epithelial repair, and barrier integrity	Moderate–high	[141]
	Minimally processed WPC versus conventional WPC	Animal study (preterm pig model)	Minimally processed WPC improved gut structure and barrier integrity versus conventional WPC	Preservation of heat-sensitive whey bioactives supporting epithelial maturation and barrier function	Moderate–high	[150]
Antioxidant/Anti-inflammatory Activity of Whey Proteins	Whey proteins	Clinical study (sedentary subjects)	Increased GSH in mononuclear cells	Increased glutathione (GSH) synthesis via cysteine/methionine supply	Moderate–high	[38]
	Whey proteins	Animal study (mice)	Increased lymphocytes count and GSH level in liver; inhibits hydroperoxide and ROS in leukocytes and liver	Increased glutathione (GSH) synthesis via cysteine/methionine supply	Moderate–high	[35]
	Lactoferrin	Animal study (multiple mouse hepatitis models)	Reduced liver injury and inflammation and improved survival in hepatitis models	Upregulation of intestinal IL-11 and protective immune signaling pathways	Moderate	[151]
	Undenatured WPC vs. casein and control WPC	Animal study (C3H/HeJ mice)	Undenatured whey protein enhanced immune response and tissue glutathione levels vs. casein and control WPC	Preserved whey bioactives (e.g., immunoglobulins, BSA); supported glutathione synthesis and immune function	Moderate	[152]
	Whey-protein-enriched nutritional support	Clinical study (ICU COVID patients)	Reduced CRP increased pre-albumin and albumin, and shorter ventilation, improved survival	Improved protein intake supporting nutrition and reducing inflammation in critical illness	Moderate–high	[168]
	Whey protein + vitamin E	Randomized controlled trial (hemodialysis patients with malnutrition)	Improved malnutrition scores SGA and MIS versus control with WPs (best with WPs + vit E)	Protein and antioxidant support reducing inflammation and oxidative stress in dialysis patients	Moderate–high	[172]
	Whey vs. soy protein	Systematic review + meta-analysis of RCTs (31 trials)	Reduced IL-6 (stronger in sarcopenic/pre-frail individuals), no effect on CRP and TNF-α (soy effective on TNF- α)	Whey-mediated modulation of inflammatory signaling pathways	Moderate–high	[173]
	Whey protein	Clinical study (RCT, patients with stroke)	Decreased IL-6, TNf-α, hs-CRP, NIHSSn improved mRS, no effect on albumin, MDA	Anti-inflammatory nutritional support cytokine modulation	Moderate–high	[183]
	Whey protein	Animal study (skeletal muscle oxidative stress model)	Reduced oxidative stress injury, improved muscle morphology, and protein synthesis	SIRT1/Nrf2/HO-1 activation and AMPK/TSC2/mTOR/4EBP1 signaling	Moderate	[184]
	GMP	Animal study (mice, high-fat, high-fructose)	Reduced obesity, insulin resistance, inflammation, oxidative stress and hepatic steatosis	Improved insulin sensitivity and reduced ER stress/inflammation	Moderate	[185]
	GMP	Animal study (rats, colitis model)	Reduced colonic inflammation (colonic alkaline phosphatase activity and interleukin 1); anorexia and tissue damage	Immune cell inhibition and reduced inflammatory mediators	Moderate	[186]
	GMP	Animal study (rats)	Reduced ileal inflammation, IL-1β, TNF-α, and IL-17	Downregulation of Th17-related inflammatory response	Moderate	[187]
	GMP	Animal study (mice C57BL/6J, streptozotocin-induced T2D)	Reduced hyperglycemia, inflammation and dyslipidemia; improved insulin sensitivity	IRS-1/PI3K/Akt signaling regulation and gut microbiota modulation	Moderate	[191]
	Lactoferrin	Animal study (rats, rheumatoid arthritis model)	Reduced arthritis, hyperalgesia and TNF-α, increased IL-10	Immunomodulation and opioid-receptor-mediated analgesia	Moderate	[198]
	Lactoferrin	Animal study (DBA/1 mice, *Staphylococcus aureus* septic arthritis)	Reduced local articular inflammation in autoimmune and septic arthritis	Local anti-inflammatory action without promoting bacterial survival	Moderate	[199]
Antimicrobial Modes of Action of Whey-Protein-Derived Peptides	Lactoferricin	Human gastric digestion study	Generation of antimicrobial lactoferricin peptides in stomach	Pepsin-mediated lactoferrin hydrolysis producing bioactive antimicrobial peptides	Moderate–high	[226]
	Lactoferricin B and lactoferrampin	In vitro (disrupting membrane integrity)	Disrupted membranes of *Candida albicans* and *E. coli*	Membrane permeabilization and structural disruption	Moderate–high	[229]
	Lactoferrampin	Structural/membrane interaction study	Antimicrobial interaction with bacterial membranes	Two-step antimicrobial mechanism: initial electrostatic attraction via positively charged *C*-terminus followed by *N*-terminal α-helix binding to the bacterial lipid bilayer	Moderate–high	[230]
	α-lactalbumin-digested peptides	In vitro (bacterial growth inhibition)	Bactericidal activity mainly against Gram-positive bacteria	Release of antimicrobial peptide domains after proteolysis	Moderate	[231]
	β-lactoglobulin-digested peptides	In vitro (bacterial growth inhibition)	VAGTWY, AASDISLLDAQSAPLR, IPAVFK, VLVLDTDYK peptides had bactericidal activity against Gram-positive bacteria; modified peptide extended activity to Gram-negative	Proteolytic release and sequence-dependent membrane-targeting antimicrobial activity	Moderate	[232]
	Lactoferricin (lactoferrin antimicrobial domain)	Peptide isolation and antimicrobial study	Potent bactericidal activity against Gram-positive and Gram-negative bacteria	*N*-terminal disulfide-bonded antimicrobial domain disrupting bacterial viability	Moderate–high	[233]
	Lactoferrampin B	Structural and membrane interaction study	Membrane defects; ion leakage and strong affinity for acidic phospholipids in bacterial membrane models	α-helical peptide insertion and electrostatic interaction with acidic bacterial membranes	Moderate–high	[234]
	Lactoferrin and lactoferricin	Antimicrobial peptide/LPS interaction study	LPS release; membrane blister formation and bactericidal activity against Gram-negative bacteria	Outer membrane disruption through LPS binding and membrane destabilization	Moderate–high	[235]
	Lactoferricin-derived peptides	Antimicrobial peptide/LPS interaction study	Antimicrobial activity associated with bovine-derived and chimeric peptides	Cationic arginine-rich residues bind negatively charged LPS, disrupting membranes and promoting tryptophan-mediated hydrophobic interactions with lipid A	Moderate	[236]
	Lactoferricin-derived peptide	Membrane permeabilization study	Cytoplasmic membrane depolarization; loss of pH gradient and bactericidal activity against *E. coli*	Inner membrane permeabilization causing transmembrane ion flux disruption	Moderate	[237]
	Lactoferricin B	Antimicrobial/macromolecular synthesis study	Inhibited DNA, RNA, and protein synthesis in *E. coli* and *B. subtilis*	Intracellular targeting impairing bacterial macromolecular synthesis	Moderate–high	[238]
	Lactoferrin and cyclic lactoferricin	In vitro (human fibroblast)	Reduced HCMV antigen expression; viral entry and infectious viral progeny	Inhibition of viral entry into host fibroblasts	Moderate	[239]
	Lactoferrin and lactoferricin B	In vitro (monocytic cell study)	Suppressed endotoxin-induced IL-6 production; lactoferricin B was the strongest inhibitor	Inhibition of LPS-triggered inflammatory signaling in monocytic cells	Moderate	[242]
	LF11 peptide (lactoferrin-derived)	Structural and biophysical study	Endotoxin neutralization and antimicrobial activity against Gram-negative bacteria	Defined amphipathic structure enabling electrostatic and hydrophobic interaction with LPS	Moderate	[244]
	Lactoferricin	In vitro (human fibroblast)	Reduced infectivity and host cell penetration of *Toxoplasma gondii* and *Eimeria stiedai* sporozoite; improved survival outcomes	Direct inhibition of parasite invasion and infectivity	Moderate–high	[245]

## Data Availability

No new data were created in this study. Data sharing is not applicable to this article.
